# Supplementary material for: Inhibitory Properties of Cinnamon Bark Oil against Postharvest Pathogen Penicillium digitatum In Vitro
Source: J Fungi (Basel). 2024 Mar 26;10(4):249. doi: 10.3390/jof10040249 (PMC11051492; doi:10.3390/jof10040249)
Supplement: Supplementary file 1 [file jof-10-00249-s001.zip › Supplementary Materials.pdf]

Inhibitory Properties of Cinnamon Bark Oil against Postharvest Pathogen Penicillium digitatum In Vitro

Ting Zhou, Jingjing Pan, Jingjing Wang, Qinru Yu, Pengcheng Zhang, Tongfei Lai*

College of Life and Environmental Science, Hangzhou Normal University, Hangzhou 310036, China; zt20100061@hznu.edu.cn (T.Z.); 2022111010065@stu.hznu.edu.cn (J.P.); 2021111010050@stu.hznu.edu.cn (Q.Y.); zpc604@hznu.edu.cn (P.Z.)

* Correspondence: laitongfei@hznu.edu.cn

Supplementary Materials:

Supplementary tables

Table S1 The information of primer pairs used in this study

Table S2 The information of all identified transcripts by transcriptomic analysis in *P. digitatum* with and without CBO treatment

Table S3 The expression changes of 1082 DEGs respond to CBO treatment in *P. digitatum*

Supplementary figures

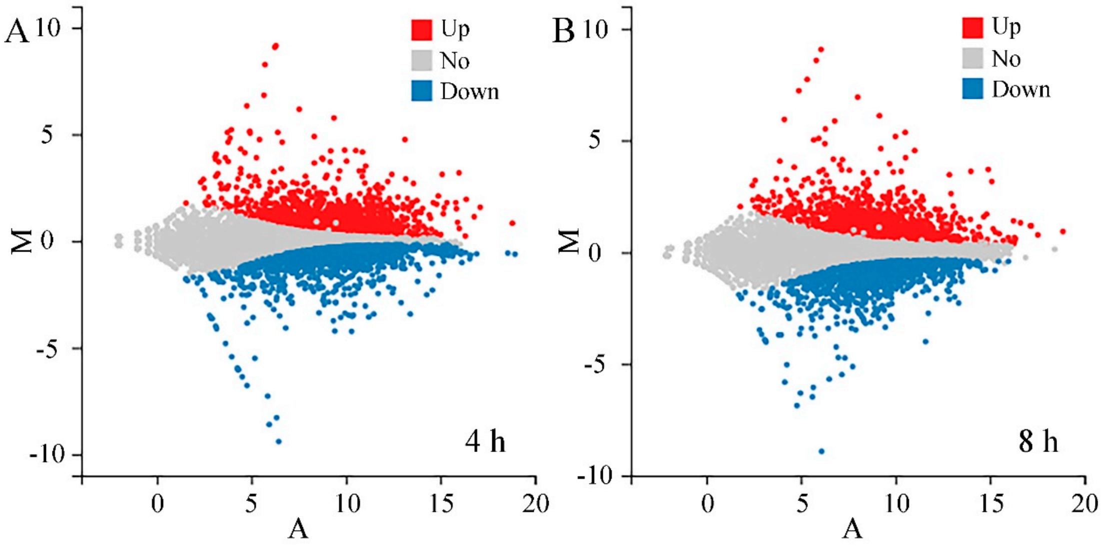

Figure S1 The MA plot of DEGs acquired from 4 h (A) and 8 h (B) results. Each point indicates a DEG, and red, grey and blue color respectively represents up-regulated, non-regulated and down-regulated. M stands for minus subtraction of log values is equivalent to the log of the ratio and on the x axis, the A stands for average.

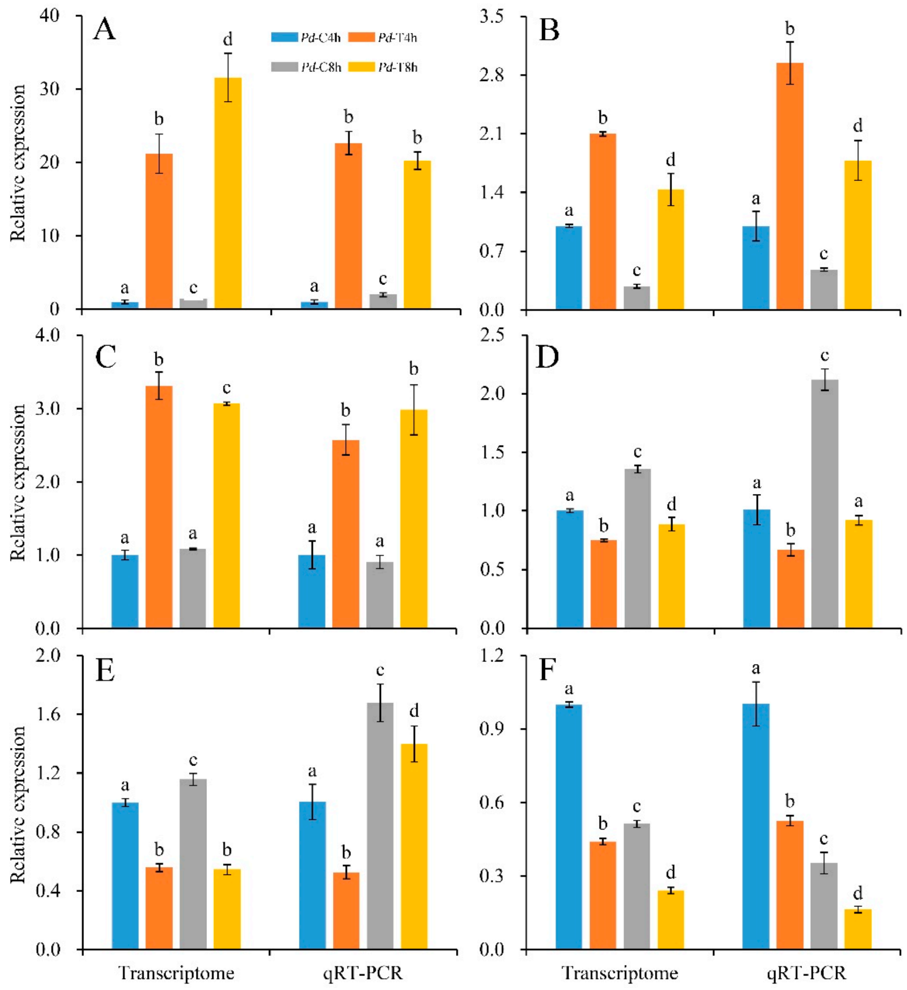

Figure S2 The relative expression levels of randomly selective DEGs in control and treatment groups acquired from the qRT-PCR and transcriptomic results. (A) to (F) show the relative expression levels of *G1* to *G6,* respectively. The detailed information of G1 to G6 is shown in Table S1. Bars indicate the standard deviation of the means. Lowercase letters a and b indicate significant differences at $p < 0.05$ based on Student's *t*-test for different detection approaches.

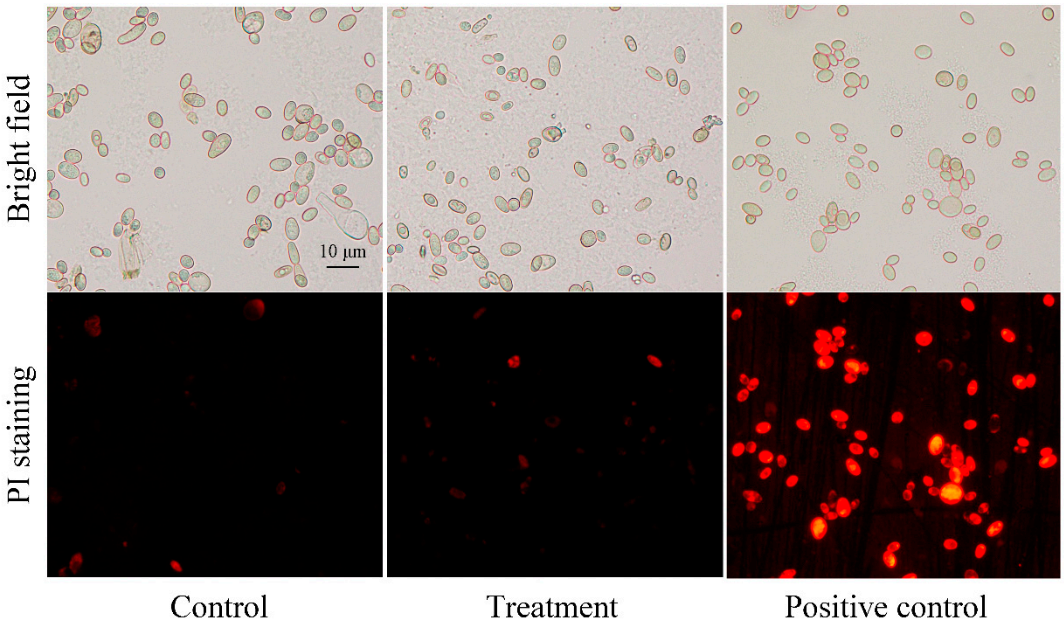

Figure S3 Effect of CBO on the membrane integrity of *P. digitatum* spores. Spores were cultured with or without 0.03% CBO treatment for 4 h. Half of CBO-treated spores were incubated in boiling water for ten minutes (as positive control). Then, all samples were stained with propidium iodide.
